# Optimal leaf water status regulation of plants in drylands

**DOI:** 10.1038/s41598-019-40448-2

**Published:** 2019-03-06

**Authors:** Gregor Ratzmann, Liubov Zakharova, Britta Tietjen

**Affiliations:** 1 Freie Universität Berlin, Institute of Biology, Altensteinstraße 34, 14195 Berlin, Germany; 2 Freie Universität Berlin, Dahlem Centre of Plant Sciences, 14195 Berlin, Germany; 3 Department of Ecosystem Modelling, University of Göttingen, Büsgenweg 4, 37077 Göttingen, Germany; 4 Berlin Brandenburg Institute of Advanced Biodiversity Research (BBIB), 14195 Berlin, Germany

## Abstract

Leaf water potential regulation is a key process in whole plant and ecosystem functioning. While low water potentials induced by open stomata may initially be associated with greater CO_2_ supply and a higher water flux from the rhizosphere to the canopy, they also inhibit cell growth, photosynthesis and ultimately water supply. Here, we show that plants regulate their leaf water potential in an optimal manner under given constraints using a simple leaf water status regulation model and data from a global dryland leaf water potential database. Model predictions agree strongly with observations across locations and species and are further supported by experimental data. Leaf water potentials non-linearly decline with soil water potential, underlining the shift from maximizing water supply to avoiding stress with declining water availability. Our results suggest that optimal regulation of the leaf water status under varying water supply and stress tolerance is a ubiquitous property of plants in drylands. The proposed model moreover provides a novel quantitative framework describing how plants respond to short- and long-term changes in water availability and may help elaborating models of plant and ecosystem functioning.

## Introduction

Leaves form an important component in the nexus of the terrestrial water and carbon cycle. They are the loci of CO_2_ uptake and act as controlling valves for the water flux through the entire plant. Within this cascade of uptake, transport and exchange, the regulation of a plants leaf water status is a key process because it is intrinsically linked to many whole-plant functioning processes. These processes include stomatal conductance and thus CO_2_ uptake^1^, canopy water supply^2^, xylem functioning^3^ and the growth of cells and ultimately organs^4^. Being able to understand and quantitatively describe how a plant regulates its leaf water status in response to changing water availability is therefore a key element in understanding and describing whole plant functioning.

The links between leaf water status regulation and other processes are complex and best considered sequentially. When a plant opens its stomata to take up carbon dioxide it inevitably accepts a loss of water through transpiration. The consequent decline in leaf water content leads to a decline in the cellular pressure and osmotic potentials and thus in the bulk leaf water potential ψ_L_, which we define as the key variable describing leaf water status. This decline in turn initiates an intercellular water flux through xylem conduits from the roots to the leaves^5^. Considering the whole pathway from the soil to the atmosphere water thus follows declining water potentials which gradually drop from the rhizosphere to the leaves^3,5^. This water flux is commonly described by Darcy’s Law or in analogy to an electric current^2,6^ and can be formalized as

1 $$Q=k|{\psi }_{L}-{\psi }_{S}|$$

Here, Q (m^3^ s^−1^ plant^−1^) is the total water flux through a plant per time, k (m^3^ s^−1^ MPa^−1^ plant^−1^) is the plant’s bulk conductance to liquid water, ψ_L_ (MPa) is the plants bulk leaf water potential, and ψ_S_ (MPa) is the bulk plant available soil water potential. The absolute value of the gradient ψ_L_ − ψ_S_ is taken because leaf and soil water potentials are smaller than 0 and ψ_L_ is typically lower than ψ_S_ when the plant actively controls transpiration.

Everything else being equal on the right side of Eq. (1) it predicts that the water flux from soil to leaves should increase and hence improve the leaf water supply with decreasing ψ_L_. Moreover, stomatal opening, which allows more CO_2_ to enter the leaves, will lead to lower ψ_L_ through transpiration. Low leaf water potentials in mesophyll cells initiate stomatal closure^7^ and thus mediate the positive effect on CO_2_ uptake. At the same time, a low leaf water potential first inhibits overall growth processes^4,8,9^, because cell wall expansion and consequently cell division is directly dependent on cell turgor pressure. Further declining leaf water potentials then start inhibiting photosynthesis^10^ and lead to turgor-induced stomatal closure^7^, which will reduce the leaf-level CO_2_ supply. Moreover, a decline in leaf water potentials corresponds to declining xylem water potentials throughout the whole plant^5,6^. This, in turn, brings about a rapid, non-linear drop in xylem hydraulic conductance caused by xylem embolism, which will also lead to a decline of k in Eq. (1), ultimately leading to a decline in canopy water supply^3,11^.

The plant is consequently faced with the challenge of, on the one hand, allowing the leaf water potential ψ_L_ to drop to a point where it can still take up CO_2_ and maintain a water flux from the soil to the leaves, while, on the other hand, avoiding leaf water potentials that will inhibit its growth and assimilation and lead to a disruption of the canopy water supply^12,13^. This challenge has to be solved by each plant for a given structural and functional configuration^13^, such as leaf to root area ratio, plant height, and xylem conductance, and for variable environmental constraints^14^, such as air vapor pressure deficit or irradiation. The means by which a plant can regulate its leaf water potential are stomatal opening and closure and accumulation of osmolytes in leaf cells.

In studies of the leaf and soil water potential relationship the approximation is often made that predawn leaf water potential (ψ_pd_, ψ_L_ at predawn) is a close proxy for soil water potential and that leaf water potentials at midday (ψ_m_, ψ_L_ at midday) are a proxy for ψ_L_^15^. We will use the approximations ψ_L_ ≈ ψ_m_ and ψ_S_ ≈ ψ_pd_ throughout this study. In fact, a plant rapidly reduces its ψ_m_ as the soil dries and ψ_pd_ declines^15–17^. This relationship between ψ_m_ and ψ_pd_ has been described using linear regressions^15^, however, without satisfyingly describing the initial rapid drop of ψ_m_ with declining ψ_pd_. The decline of ψ_m_ continues until a critical point ψ_crit_ is reached, where ψ_m_ = ψ_pd_^16^, at which the driving gradient from soil to leaves (Eq. (1)) has vanished and stomata are entirely closed^16,18^. Moreover, maintaining ψ_m_ above ψ_crit_ as long as ψ_pd_ > ψ_crit_ prevents the plant from embolism in leaf and stem conduits^19,20^. Values of 0 > ψ_m_ > ψ_crit_ thus form the range of water potentials which are under the active control of the plant.

Despite considerable advancements in quantifying leaf water status and stomatal regulation^12,15,16^, a quantitative description of the dilemma that a plant is facing when regulating its leaf water potential is still missing.

Here, we propose a quantitative description of leaf water status regulation under drying soil conditions using a simple model and hypothesize that plants regulate their leaf water potential optimally based on the described tradeoff rather than on a purely descriptive linear regression^15^. We test the model using a database of concurrent observations of ψ_pd_ and ψ_m_ for global drylands. We additionally test the model against observations of ψ_pd_ and ψ_m_ during a soil drying experiment for several woody species conducted by Meinzer *et al*.^16^. This test additionally provides a benchmark of the model’s applicability beyond drylands.

## Theoretical Framework

**Figure 1:**
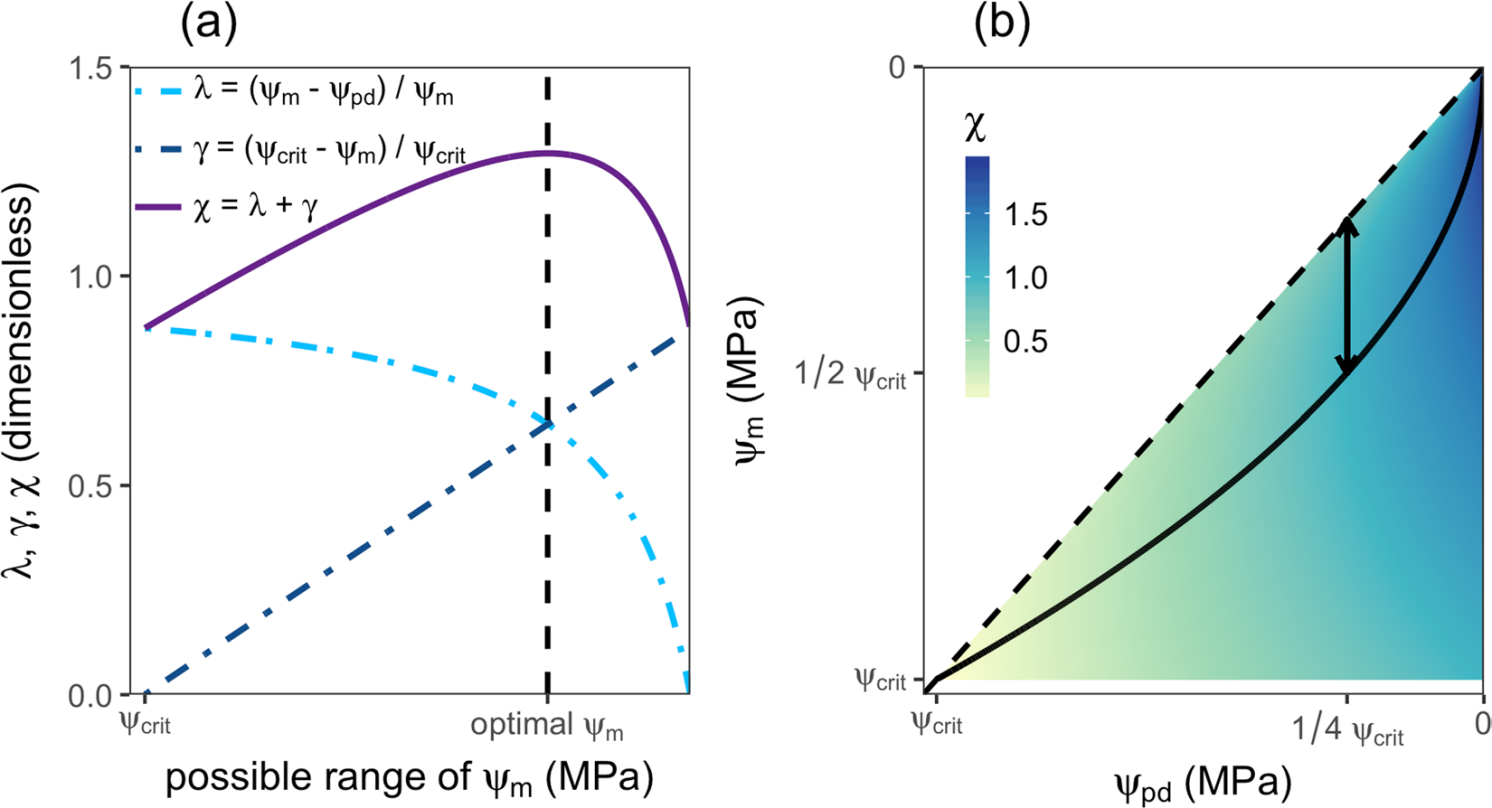
Schematic drawing of the described leaf water status optimization. (**a**) shows an example of how the optimal leaf water potential ψ_m_ is found for a given fixed ψ_pd_ over its entire possible range of leaf water potentials (ψ_crit_ < ψ_m_ < 0). Stress induced by low water potentials (criterion γ) is least when ψ_m_ approaches zero (high γ) while the water potential gradient maximization criterion λ reaches its maximum as ψ_pd_ approaches the critical water potential ψ_crit_. The optimum leaf water potential for the given ψ_pd_ is found where χ has its maximum (black dashed line) and λ and γ intersect. (**b**) shows an example of the optimal leaf water potential (approximated by midday water potential, ψ_m_, black solid line) for every possible soil water potential (approximated by predawn water potential, ψ_pd_) (ψ_crit_ < ψ_pd_ < 0). The coloring represents the optimization criterion χ for any given ψ_pd_: the optimal leaf water potential ψ_m_ for any given soil water potential ψ_pd_ is found where χ is highest (example for a fixed soil water potential in (**a**)); the dashed black line represents the one to one line on which the gradient of Eq. (1) vanishes; the arrow represents the point where the rhizosphere to canopy water potential gradient is maximal and at which ψ_pd_ = 1/4 ψ_crit_ and ψ_m_ = 1/2 ψ_crit_.

A drop in a plant’s leaf water potential is an inevitable consequence of stomatal opening and transpiration. However, low leaf water potentials come at the cost of low growth and assimilation efficiencies and potential negative effects on the leaf supplying conduits. We suggest that this tradeoff can be described as follows (Fig. 1). The first criterion, namely that the plant needs to maximize the gradient ψ_m_ − ψ_pd_ and thus canopy water and CO_2_ supply, can be expressed as

2 $$\lambda =\frac{{\psi }_{m}-{\psi }_{pd}}{{\psi }_{m}}$$

The second criterion, namely to keep ψ_m_ as high as possible, is based on the fact that the more ψ_m_ approaches ψ_crit_, the more desiccation-stressed this leaf is and can be written as

3 $$\gamma =\frac{{\psi }_{crit}-{\psi }_{m}}{{\psi }_{crit}}$$

Relating both distances (ψ_m_ − ψ_pd_ and ψ_crit_ − ψ_m_) to a reference value (ψ_m_ in Eq. (2) and ψ_crit_ in Eq. (3)) has the effect that both criteria λ and γ are made dimensionless. An optimal leaf water potential for any soil water potential should thus maximize

4 $$\chi =\lambda +\gamma $$

with the underlying simple assumption that both criteria are equally weighted, and consequently suffice

5 $$\frac{d\chi }{d{\psi }_{m}}=0$$

The leaf water potential which satisfies Eq. (5) for any ψ_pd_ has two solutions, one with a positive sign and one with a negative sign. The latter is appropriate in this case and given by

6 $${\psi }_{m}=-\sqrt{{\psi }_{crit}{\psi }_{pd}}$$

Equations (2–6) predict that for any given ψ_pd_ there is one optimal ψ_m_ (Fig. 1a). Increasing ψ_m_ beyond this value would result in a reduced stress level due to low water potentials at the cost of reduced water and CO_2_ supply. On the other hand, further reducing ψ_m_ at the given ψ_pd_ would result in a dominance of the negative effects (γ), such as growth inhibition and reduced canopy water supply, and thus lead to a smaller χ. Further, Eq. (6) predicts a rapid decline of ψ_m_ with decreasing ψ_pd_ under well-watered conditions, which becomes more gradual and linear as the soil keeps drying (Fig. 1b). This pattern indeed is often observed when studying leaf water potential responses to soil drying^16,17^.

Besides predicting the response of ψ_m_ to changing ψ_pd_ the optimal leaf water status regulation model makes two specific predictions. Firstly, the distance between ψ_m_ and ψ_crit_ should monotonically increase as ψ_pd_ increases and thus stress induced by low leaf water potentials should decrease. The second specific prediction concerns the gradient between the rhizosphere and the canopy (ψ_m_ − ψ_pd_), which in most instances is the strongest driver of upward water flux through a plant (vertical distance between the solid and the dashed line in Fig. 1b). According to the optimal leaf water status regulation model, this gradient is predicted to be highest at a predawn water potential of ψ_pd_ = 1/4 ψ_crit_ and correspondingly at midday leaf water potentials of ψ_m_ = 1/2 ψ_crit_ (d(ψ_m_ − ψ_pd_)/dψ_pd_ = 0, arrow in Fig. 1b). This implies (everything else being equal) that transpiration should be maximal at intermediate values of ψ_m_, which indeed has been shown to be the case^21^.

The proposed leaf water status regulation model predicts leaf water potentials under the active control of the plant, that is, ψ_m_ > ψ_crit_. ψ_m_ may, however, fall below ψ_crit_ induced by, e.g., cuticular transpiration. As the proposed model is concerned with leaf water status regulation, those values induced by uncontrolled transpiration are beyond the scope of the model.

We acknowledge that this minimal model lumps several processes, such as the relationship between xylem pressure and conductance, into two simple optimization criteria. Yet, we believe that this helps analytically understanding the model without the need to fit many parameters.

## Results

### Comparison of predictions with the database

**Figure 2:**
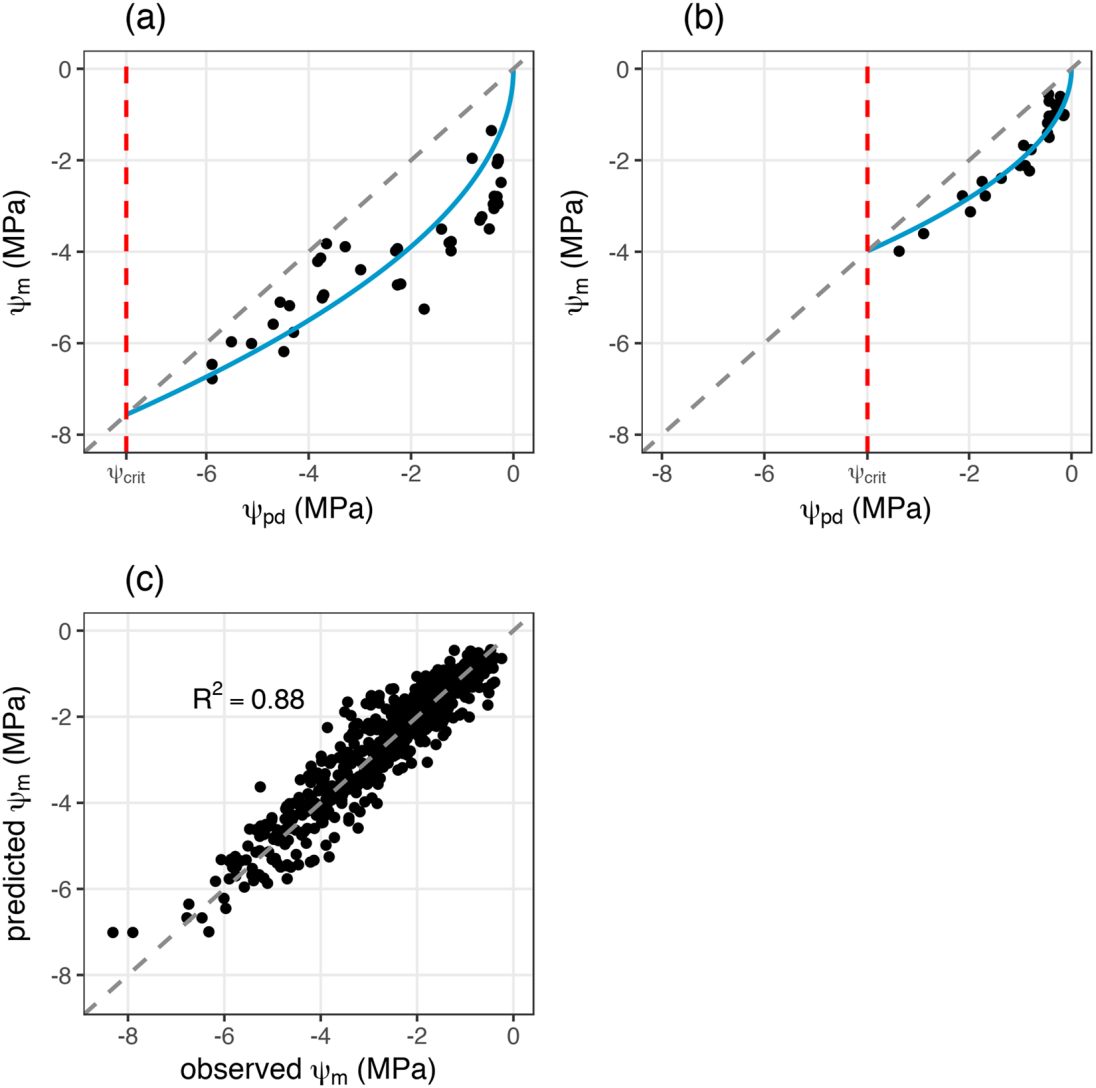
Comparison of predictions of the optimal leaf water status regulation model and measured data. Examples of the optimal leaf water status regulation model (blue line) fitted (**a**) to measured data of *Acacia berlandieri*^22^ and (**b**) to measured data of for *Arbutus unedo*^33^, grey lines in (**a,b**) are the one to one lines where ψ_pd_ = ψ_m_ and the red lines indicate the position of the estimated ψ_crit_; (**c**) comparison of observed ψ_m_ against predicted ψ_m_ for all data in the database, the grey dashed line is the one to one line.

The optimal leaf water status regulation model is able to reproduce the initial rapid drop of ψ_m_ for high values of ψ_pd,_ which becomes more gradual with declining ψ_pd_, here shown for two exemplary datasets of *Acacia berlandieri* (Fig. 2a) and of *Arbutus unedo* (Fig. 2b), which both homogeneously cover the entire operating range of ψ_pd_ until ψ_crit_.

The overall agreement of model predictions and observations for each dataset in the dryland database is very good, with most of the variance in the data being captured by the model (Fig. 2c, R^2^ = 0.88) and no systematic bias being apparent from the comparison. Individual model R^2^ values are variable but generally in the range of the overall R^2^ value (Supplementary Fig. S1, Table S2).

### Comparison of predictions with experimental data

To perform an independent test of the model’s ability to explain observed concurrent reductions of ψ_pd_ and ψ_m_ we applied the model to an experimental dataset^16^ using the experimentally determined and the estimated ψ_crit_ (see Methods) as input to the model.

**Figure 3:**
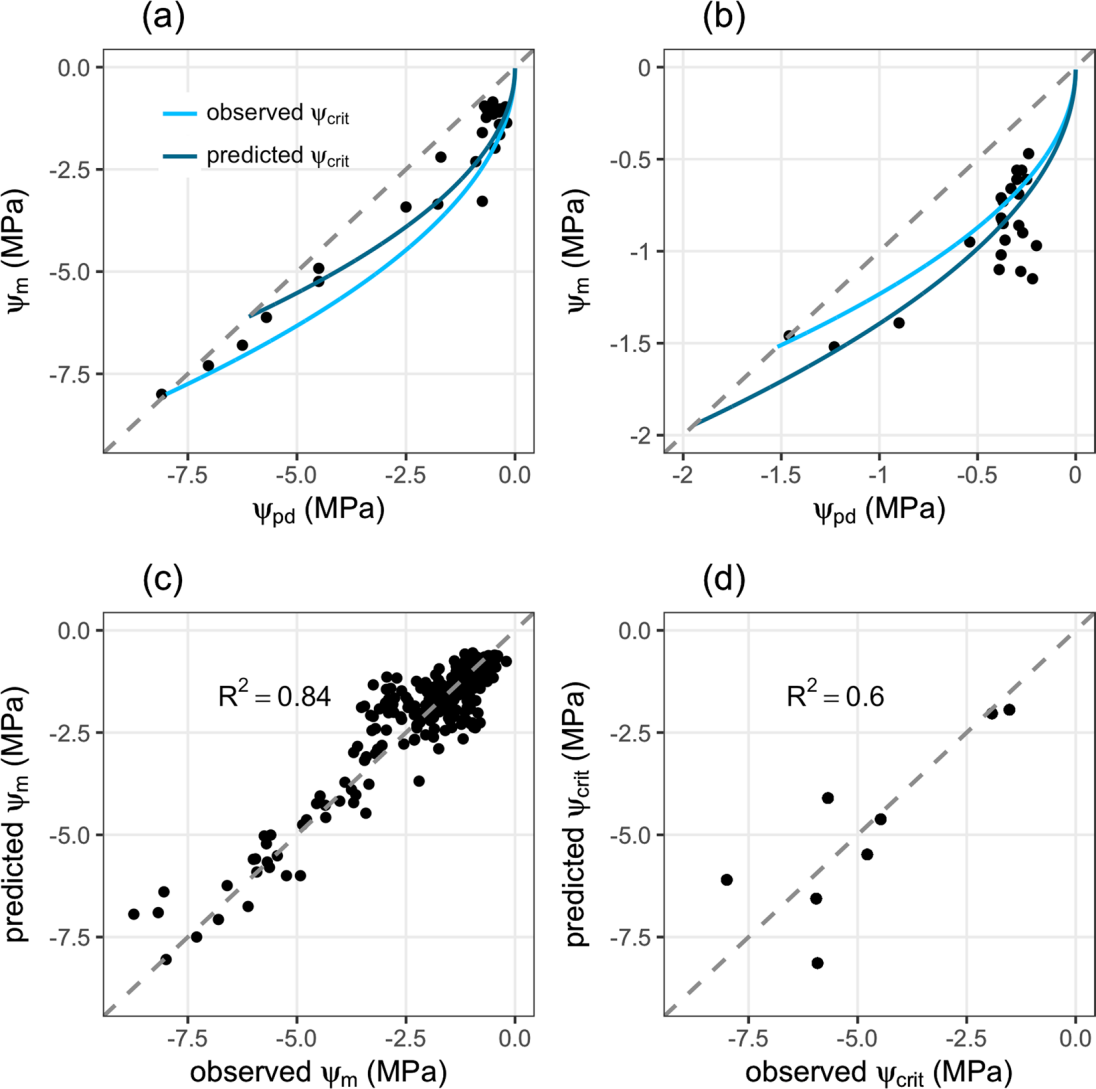
Comparison of predictions of the optimal leaf water status regulation model with measured data^16^. Examples of the optimal leaf water status regulation model (solid lines) calculated (**a**) for *Ceanothus cuneatus* and (**b**) *Salix scouleriana* based on measured and estimated ψ_crit_ values, grey lines in (**a,b**) are the one to one line where ψ_pd_ = ψ_m_. The examples were chosen for two species representing ψ_crit_ on the upper and lower end, respectively; (**c**) shows the comparison of observed ψ_m_ against predicted ψ_m_ (based on measured ψ_crit_) for all data in the experiment, the grey dashed line is the one to one line; (**d**) shows a comparison of measured ψ_crit_^16^ (observed) and ψ_crit_ estimated using residual minimization procedures (see Methods), the grey dashed line is the one to one line.

The model reproduces the overall pattern of concurrent reduction of ψ_m_ with ψ_pd_ well. The underlying functional response of ψ_m_ to ψ_pd_ is shown exemplarily for *Ceanothus cuneathus* (Fig. 3a) and *Salix scouleriana* (Fig. 3b), which are in the lower and upper end of ψ_crit_, respectively, of the experimental data. Further comparisons are given in Supplementary Table S1 and Supplementary Figure S4. Also, the overall agreement of model predictions of ψ_m_ with observed data is very good (Fig. 3c, R^2^ = 0.84) and no bias in model predictions can be observed.

To further test the applicability of the model fitting procedure we compared ψ_crit_ predicted by minimizing the residuals of the model fitted to the experimental data to experimentally determined ψ_crit_ values (Fig. 3d). The general agreement between both, estimated and observed ψ_crit_ is good (R^2^ = 0.6), although particularly for lower values of ψ_crit_ our model predictions deviate strongly from observations; however, without any bias towards lower or higher values.

### Specific predictions of stress avoidance and hydraulic functioning

**Figure 4:**
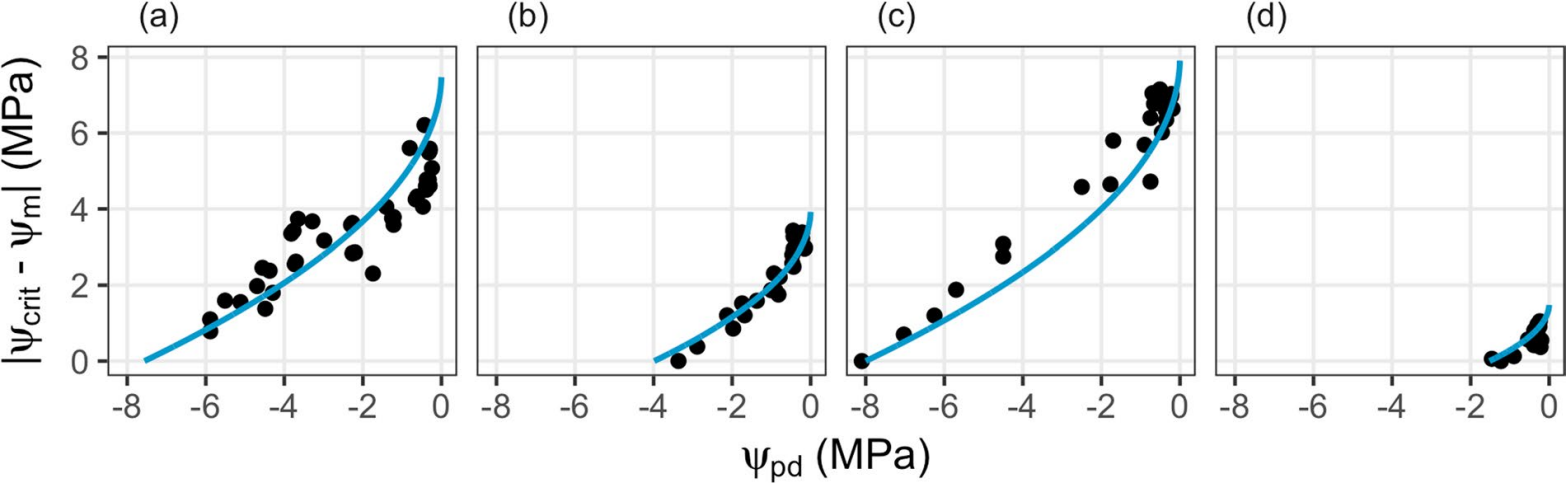
Examples of the specific model prediction of stress avoidance, which states that the distance ψ_crit_ − ψ_m_ should increase as ψ_pd_ increases. Examples taken are as in Fig. 2 (database, (**a**) and (**b**)) and 3 (dry-down experiment, (**c,d**)): (**a**) *Acacia berlandieri*, (**b**) *Arbutus unedo*, (**c**) *Ceanothus cuneatus* and (**d**) *Betula scouleriana*. Points are observations, blue lines are model predictions. Model predictions are based on estimated ψ_crit_ ((**a,b**) and measured ψ_crit_ (**c,d**)).

The proposed concept specifically predicts that the effects of stress induced by low leaf water potentials should monotonically decrease as ψ_pd_ increases. We tested this prediction for two example species from the database (as in Fig. 2a,b) and two example species from the data of the dry-down experiment (as in Fig. 3a,b). The results indeed show that all examples follow the predicted non-linear pattern of stress avoidance closely (Fig. 4, Supplementary Fig. S3a): irrespective of the range of water potentials, ψ_crit_ − ψ_m_ declines with declining ψ_pd_, with the strongest change in ψ_crit_ − ψ_m_ at low stress levels.

**Figure 5:**
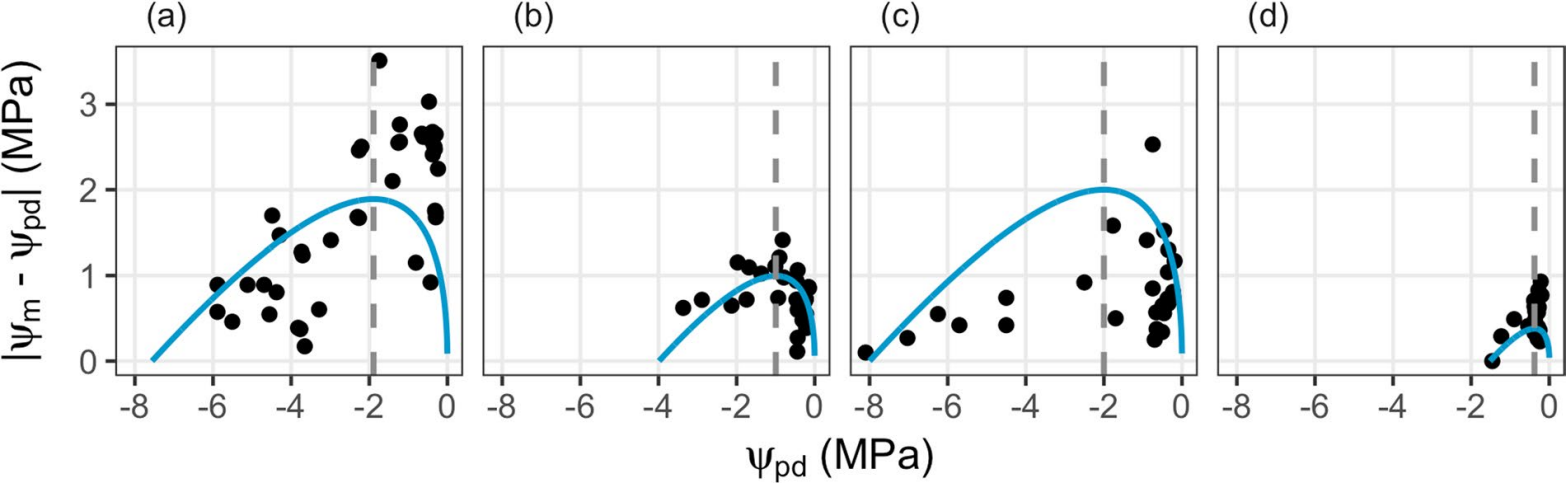
Examples of the second specific model prediction stating that the water potential gradient responsible for water flux from the rhizosphere to the canopy, ψ_m_ − ψ_pd_, should increase as ψ_pd_ decreases until a certain point ψ_pd_ = 1/4 ψ_crit_ from which on this gradient decreases again until it reaches ψ_crit_. Examples taken are as in Fig. 2 (database, (**a,b**)) and 3 (dry-down experiment, (**c**) and (**d**)): (**a**) *Acacia berlandieri*, (**b**) *Arbutus unedo*, (**c**) *Ceanothus cuneatus* and (**d**) *Betula scouleriana*. Points are observations, blue lines are model predictions. Model predictions are based on estimated ψ_crit_ ((**a,b**)) and measured ψ_crit_ ((**c,d**)).

The second specific model prediction states that the driving gradient of water flux from the rhizosphere to the canopy (Eq. (1)) ψ_m_ − ψ_pd_ should be maximal at ψ_pd_ = 1/4 ψ_crit_ and ψ_m_ = 1/2 ψ_crit_. We compared this hypothesized relationship to the observed pattern (Fig. 5). The observed pattern is less clear and less strongly follows the prediction (Supplementary Fig. S3b) as compared to the first specific prediction (Fig. 4), but is nonetheless apparent: As ψ_pd_ declines, the gradient ψ_m_ − ψ_pd_ rapidly increases until ψ_pd_ reaches a certain point (Fig. 1b, dashed grey line in Fig. 5). At this point, the water potential gradient between the rhizosphere and the canopy is maximal. As ψ_pd_ further declines, the gradient ψ_m_ −ψ_pd_ decrease again, until it vanishes and ψ_pd_ = ψ_m_ = ψ_crit_. The overall shape of the response of ψ_m_ − ψ_pd_ to ψ_pd_ and its maximum thus follow the predictions made by the presented optimal leaf water status regulation model.

## Discussion

When plants take up CO_2_ they are in the dilemma of allowing their leaf water potentials to drop through transpiration while avoiding reduced growth, assimilation and hydraulic conductance through low leaf water potentials. We present a simple model, which formalizes this tradeoff and predicts how plants may optimally solve this problem. We show that optimal leaf water potential regulation indeed appears to be a ubiquitous property of plants of a dryland leaf water potential database. Experimental results moreover indicate optimal leaf water status regulation beyond drylands.

Despite the overall strong agreement between model predictions and observations the model does not capture all variance in the data and does not necessarily explain the variance for all examples better than a simple linear regression. However, as stated in the introduction, our main aim was to describe the tradeoff of leaf water status regulation on a functional rather than a statistical basis, and thus, improving the goodness of fit was not the central point of our analysis. The large overall variance in the data is the result of several factors: Besides water supply, air vapor pressure deficit (VPD) has a strong effect on leaf water potential^22^. This effect is mediated by stomatal conductance, which is proportional to the inverse square root of VPD^23^. Other factors which have an effect on ψ_m_ are irradiation as well as changes of the leaf or root area, e.g., through herbivory^24^. Results obtained from the specific model predictions (Eqs (2, 3) and Figs 4, 5) indicate that those deviations from the optimal leaf water status regulation are mainly caused by deviations from the water potential gradient maximization criterion (Eq. (2), stronger deviations of observations from predictions in Fig. 5 than in Fig. 4, Supplementary Fig. S3). This shows that stress avoidance is a strong driver of optimal leaf water status regulation. On the other hand, maximizing the leaf water supply for a specific soil water supply might not be possible or necessary in all circumstances, such as under high VPD or high irradiation. Despite those deviations, all examples show the predicted maximum of |ψ_m_ − ψ_pd_| at intermediate values of ψ_pd_, which correspond approximately to the predicted 1/4 ψ_crit_. This supports the findings of Manzoni *et al*.^21^, who found maximum transpiration rates at intermediate values of xylem water potential. Our model shows that in addition to the interplay of xylem water potential and conductance, also the gradient between leaf and soil water potential may lead to maximum transpiration at intermediate values of ψ_m_^25^.

The plant trait ψ_crit_ is the only parameter to the presented optimal leaf water status regulation model. It is the value of leaf water potentials at which stomata are entirely closed and any decline of ψ_m_ beyond this value is likely due to uncontrolled cuticular transpiration. There is evidence that maintaining leaf water potentials above this point prevents the plant from xylem embolism^19,20^. Maintaining ψ_m_ above ψ_crit_ can, e.g., be achieved by reducing stomatal conductance or by reducing the leaf area. Leaf water potentials around ψ_crit_ maintained over longer periods, such as days or weeks, are consequently likely to lead to a partial or complete canopy defoliation^19^. Knowing ψ_crit_ can thus help predicting the way how a plant adapts to dry periods: plants that have a comparably high ψ_crit_ and thus a narrow operating space for their ψ_m_ must balance this through structural adjustments, such as reducing their leaf area^26^. They are more conservative and close their stomata earlier, which will also have a positive effect on soil water availability. On the other hand, plants with lower ψ_crit_ will rather adapt physiologically to a drying soil through osmotic adjustments at the leaf level^24^. Although we assume ψ_crit_ to be fixed over time in this study, plants in fact may adjust this trait by following water availability^27,28^. Our assumption of plants optimizing their leaf water potential in response to changing soil water potential is therefore rather a matter of hours to days, whereas the adjustment of ψ_crit_ happens over weeks or even months^27^. ψ_crit_ can thus be expected to be variable over longer time periods, but only within a plant’s structural (leaf to root area ratio, leaf morphology, xylem anatomy) and physiological (osmolyte supply) boundaries^24^. The values of ψ_crit_ found in this study (Supplementary Fig. S1) are partly lower than the overall minimum ψ_L_ at stomatal closure found by Martin-StPaul *et al*.^29^. However, considering that our study focused mainly on dryland species those lower water potentials at stomatal closure might be expected as dry-adaption. Moreover, they are in the range of ψ_L_ at stomatal closure found by Klein^30^.

It is likely that we have under- or overestimated ψ_crit_ for several datasets in the dryland database due to the inherent scatter in the water potential trajectories (Figs 2, 3d). Determining the water potential at full stomatal closure is challenging due to the asymptotic nature of the response function of stomatal conductance to ψ_L_^30^ and the possible presence of cuticular transpiration. This may have led to relatively low estimates of ψ_crit_ (Fig. 2a, Supplementary Figs S1d and S5), which in some cases certainly are below the actual water potential at full stomatal closure. Moreover, if ψ_m_ is more strongly controlled by other factors than soil water potential, such as VPD, low ψ_m_ at relatively high ψ_pd_ values will affect the model fit and consequently the estimated ψ_crit_ (Fig. 3b, Supplementary Figs S4 and S5).

We generally advise that those potential issues should be considered when applying the optimal leaf water status regulation model. Ideally, the model should be applied to a dataset where both are available, trajectories of ψ_pd_ vs. ψ_m_ as well as the water potential at full stomatal closure. If only the latter is known (or a surrogate thereof, such as the leaf water potential at turgor loss^29^) the model can also be applied to simulate the response of leaf water status to soil water availability in a coupled model of plant functioning.

Using a simple model, we found strong support for optimal leaf water status regulation as a fundamental part of plant functioning. These findings contribute to the understanding of how plants regulate their whole hydraulic apparatus and how they may respond to short- and long-term drought stress. Given that our findings and the presented model describe water status regulation of leaves, which form the pivotal link between water supply and demand, it can help elaborating models of whole-plant performance and ecosystem functioning under changing environmental conditions. The proposed model provides a novel view on how plants manage their water balance and provides a way ahead to combine modeling and empirical approaches.

## Methods

### Database

We compiled a database of concurrent observations of predawn water potential ψ_pd_ and midday water potential ψ_m_ using primary data published in the peer reviewed scientific literature. The database was established as follows: Following Martínez-Vilalta *et al*.^15^ selection criteria for studies to be included were: predawn and midday water potentials were measured concurrently, plants were grown under field conditions (including agricultural fields, but not potted plants), and studies on artificial modifications of plant water potentials were excluded.

We searched Google Scholar for the terms “savanna” + “leaf water potential” as well as “dryland” + “leaf water potential”. We also followed the database compiled by Matínez-Vilalta *et al*.^15^. and extracted data points for studies listed in their literature table. All data points were extracted from the original articles using WebPlotDigitizer (https://automeris.io/WebPlotDigitizer).

We focused on studies conducted in dry environments since water potentials in water-limited environments are likely to span a wider range. An aridity index (mean annual precipitation divided by mean annual potential evapotranspiration) was used as a decision criterion whether a study site was considered dry or not. Only locations which have an aridity index smaller 0.75 were regarded as drylands^31^. To this end, only studies reporting the explicit coordinates of the study location or allowing for their reconstruction were considered. If a study reported different study locations, they were only treated separately if their distinct coordinates were available. The aridity index at a given site was then extracted using the dataset of  ^32^.

For a given site, data points were aggregated at the species level, but not on the sub-species level. If data for the same species were found for different locations, those data points were treated separately. This was deemed necessary as a variable ψ_crit_ can be an intraspecific adaption to different environments^28^. An overview of the different locations for which data were extracted is shown in Supplementary Fig. S2.

To base the analyses on a reliable basis, we only considered datasets with at least seven concurrent measurements of ψ_pd_ and ψ_m_. Additionally, to obtain a robust model fit, we only considered datasets that covered at least 25% of the distance between 0 MPa and ψ_crit_ (Supplementary Fig. S1, value *rel_range* in Supplementary Table S2). We used this post-hoc criterion to omit datasets, for which the range of available data was too small to make reliable predictions of ψ_crit_. The cut-off threshold of 25% was selected based on the observation that above this value R^2^ values of most model fits were positive, while for lower values R^2^ was often negative (Supplementary Fig. S1). We deemed an R^2^ larger 0 as a criterion for a good model fit, since this indicates that the model captures more than the variance captured by the mean. The R^2^ was calculated as 1 minus model residual sum of squares relative to the residual sum of squares to the mean. This additional post-hoc criterion resulted in a total removal of 15 datasets initially included in the analysis. The literature search and database compilation resulted in 28 publications that were included in the analysis (Supplementary Tables S2 and S3). From those studies, a total of 43 unique datasets containing 38 species were extracted. Each data set consists of on average 16 observations. The database covers a wide range of leaf water potentials stretching from −6.6 MPa to −0.03 for ψ_pd_ and from −8.3 MPa to −0.2 MPa for ψ_m_ (Fig. 2, Supplementary Table S2).

The data collected in this database span several time periods ranging from days to months and sometimes include data which were averaged over several individuals. To test the presented concept across several species over a fixed time span under controlled conditions comprising a single soil dry-down experiment, we additionally used data published in Meinzer *et al*.^16^. ψ_pd_ and ψ_m_ of eight woody species (Supplementary Table S1) where measured during the dry-down experiment until ψ_m_ equaled ψ_pd_. This was then assumed to be the critical water potential ψ_crit_. The range of water potentials covered by the experimental data is similar to the range covered by all other datasets in our new database with minimum ψ_pd_ of −8.1 MPa and maximum ψ_pd_ of −0.15 MPa, as well as minimum ψ_m_ of −8.74 MPa and maximum ψ_m_ of −0.2 MPa. For a more detailed description of the experiment see Meinzer *et al*.^16^.

### Application of the model

We fitted the model describing the optimal leaf water status regulation (Eq. (6)) to each single data set (separated by species and location) by determining ψ_crit_ that minimizes the residual sum of squares of the predicted versus the measured data. Specifically, we selected the ψ_crit_ from a range of water potentials between −15 to −0.05 MPa with a total length of 1000 numbers that minimized the residual sum of squares for each unique dataset.

We further compared ψ_crit_ values measured during the dry-down experiment^15^ to the one obtained by minimizing the residuals of our model.

Data shown for the global dryland database do not comprise the data of the dry-down experiment.

All data of the database are available in Supplementary Table S2.

## Supplementary information

Supplementary Information

S2

S3
