# Supplementary material for: Optimal leaf water status regulation of plants in drylands
Source: Sci Rep. 2019 Mar 6;9:3768. doi: 10.1038/s41598-019-40448-2 (PMC6403219; doi:10.1038/s41598-019-40448-2)
Supplement: Supplementary file 1 — Supplementary Information [file 41598_2019_40448_MOESM1_ESM.docx]

Supplementary Information

**Optimal leaf water status regulation of plants in drylands**

Authors: Gregor Ratzmann*, Liubov Zakharova and Britta Tietjen

*corresponding author, email address: [gregor.ratzmann@gmail.com](mailto:gregor.ratzmann@gmail.com)


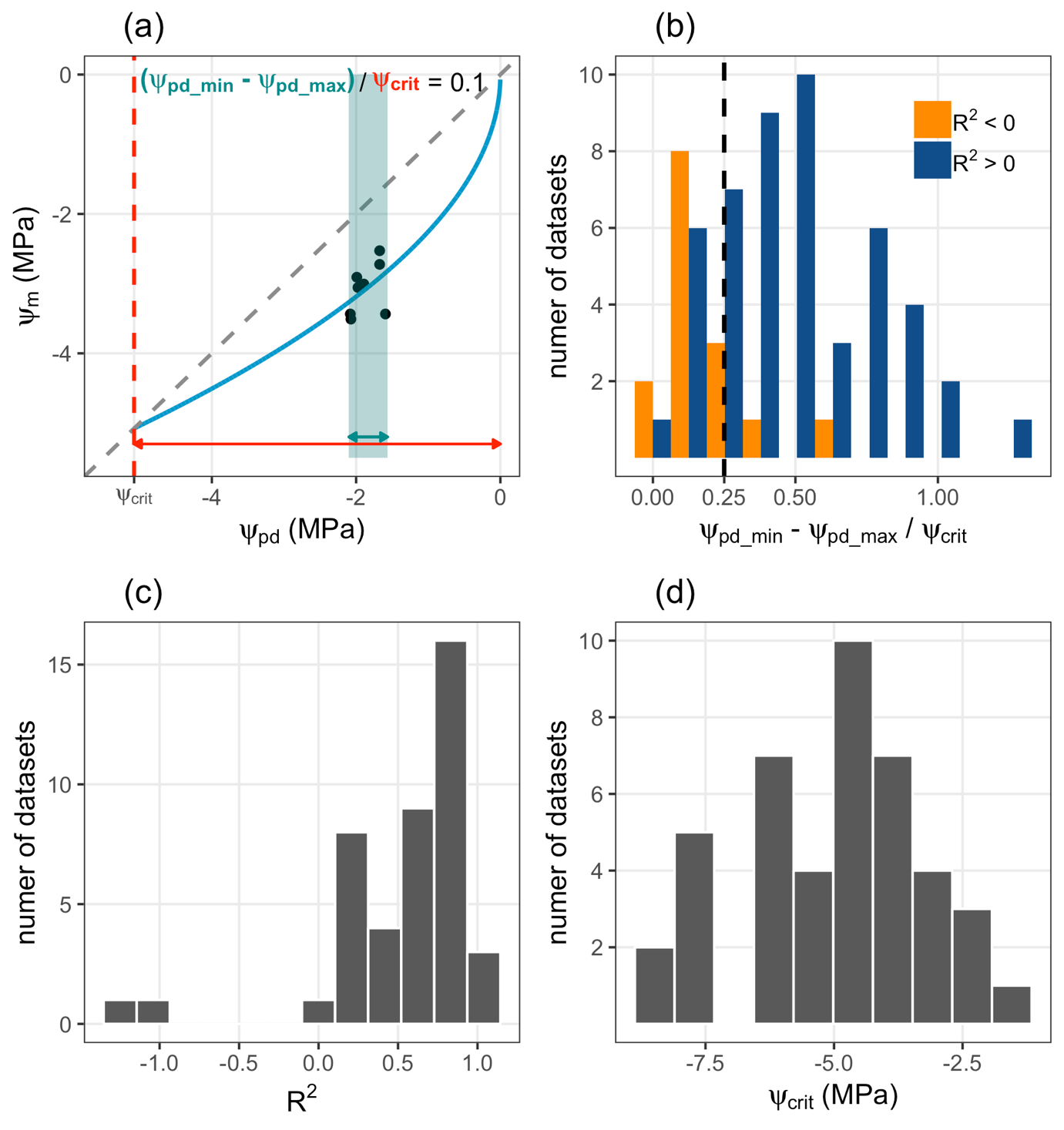


**Supplementary Figure S1** Depiction of the exclusion criterion, individual model goodness of fit and distribution of the estimated ψ_crit_ values. (a) Example plot for a dataset^1^, which does not match the criterion to be included. The range of ψ_pd_ values (cyan arrow and shaded area) is only 10 % of the distance 0 – ψ_crit_ (red arrow). The black dots are measured data and the blue solid line is the model prediction. (b) histogram of the decision criterion whether a dataset was considered in this study or whether it was disregarded. The dashed vertical line indicates the threshold: above this line all datasets were used. Note that this threshold approximately corresponds to the point from where on R^2^ values become positive. (c) Distribution of individual model R^2^ values. (d) Distribution of the estimated ψ_crit_ values for all datasets in the database.


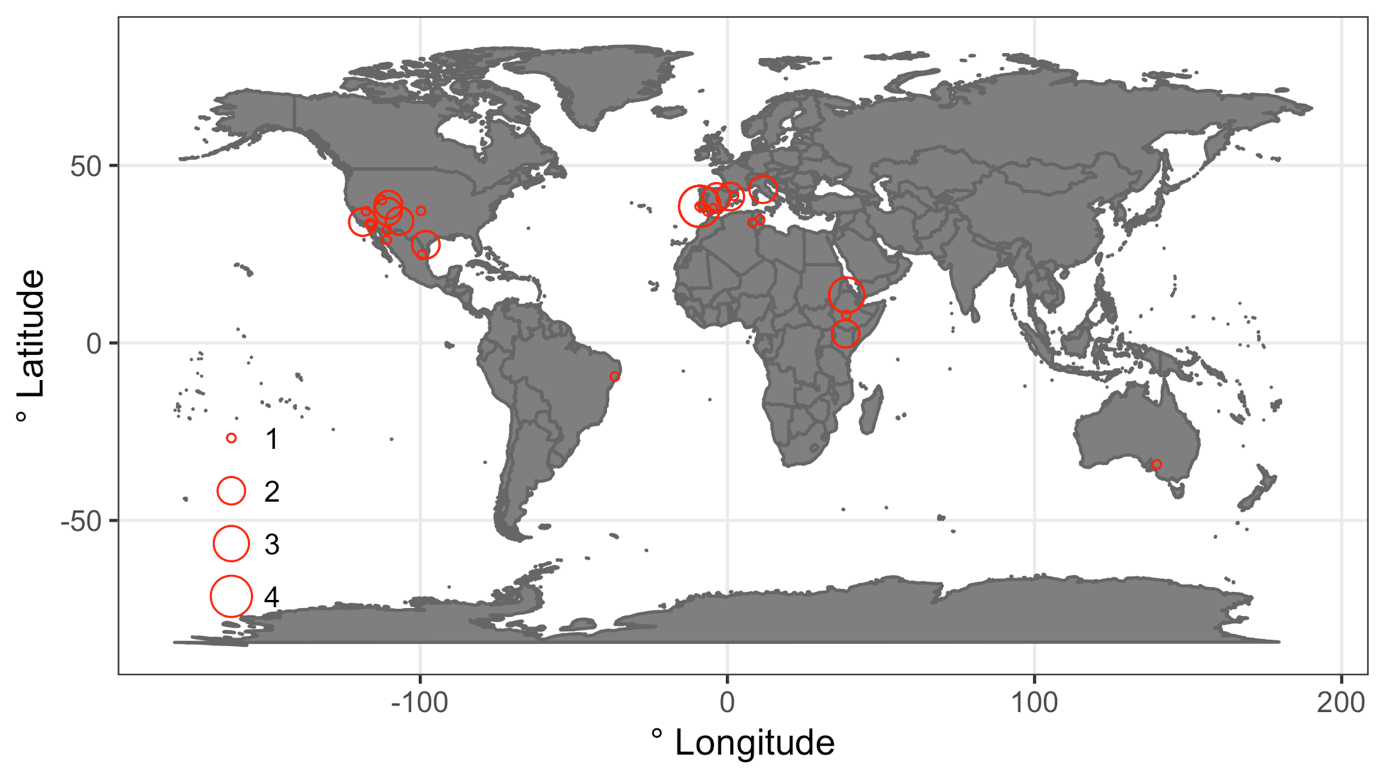


**Supplementary Figure S2** Distribution of the datasets included in the dryland database. Circle size indicates the number of datasets for a given location.

**Supplementary Table S1** Overview of the characteristics of the optimal leaf water status regulation model applied the dry-down experiment of Meinzer *et al.* ^2^. n is the number of data points of the corresponding species, measured ψ_crit_ is the measured leaf water potential where ψ_pd_ becomes equal to ψ_m_, the estimated ψ_crit_ was found using the residual minimization procedure, the R^2^ based on measured ψ_crit_ refers to ψ_m_ predicted by the optimal leaf water status regulation model using the measured ψ_crit_ as input versus the measured ψ_m_, the R^2^ based on estimated ψ_crit_ refers to ψ_m_ predicted by the optimal leaf water status regulation model using the estimated ψ_crit_ as input versus the measured ψ_m_.

| Species | n | Measured ψ_crit_ | Estimated ψ_crit_ | R^2^ based on measured ψ_crit_ | R^2^ based on estimated ψ_crit_ |
| --- | --- | --- | --- | --- | --- |
| *Betula occidentalis* | 27 | -1.92 | -2.03 | 0.55 | 0.56 |
| *Ceanothus cuneatus* | 28 | -8 | -6.1 | 0.87 | 0.92 |
| *Cerocarpus ledifolius* | 43 | -4.47 | -4.62 | 0.8 | 0.8 |
| *Heteromeles arbutifolia* | 25 | -4.78 | -5.48 | 0.8 | 0.82 |
| *Quercus garryana* | 29 | -5.92 | -8.14 | 0.46 | 0.56 |
| *Rhammus ilicifolia* | 35 | -5.68 | -4.1 | 0.68 | 0.79 |
| *Quercus douglasii* | 29 | -5.95 | -6.56 | 0.69 | 0.7 |
| *Salix scouleriana* | 24 | -1.52 | -1.94 | 0.37 | 0.52 |


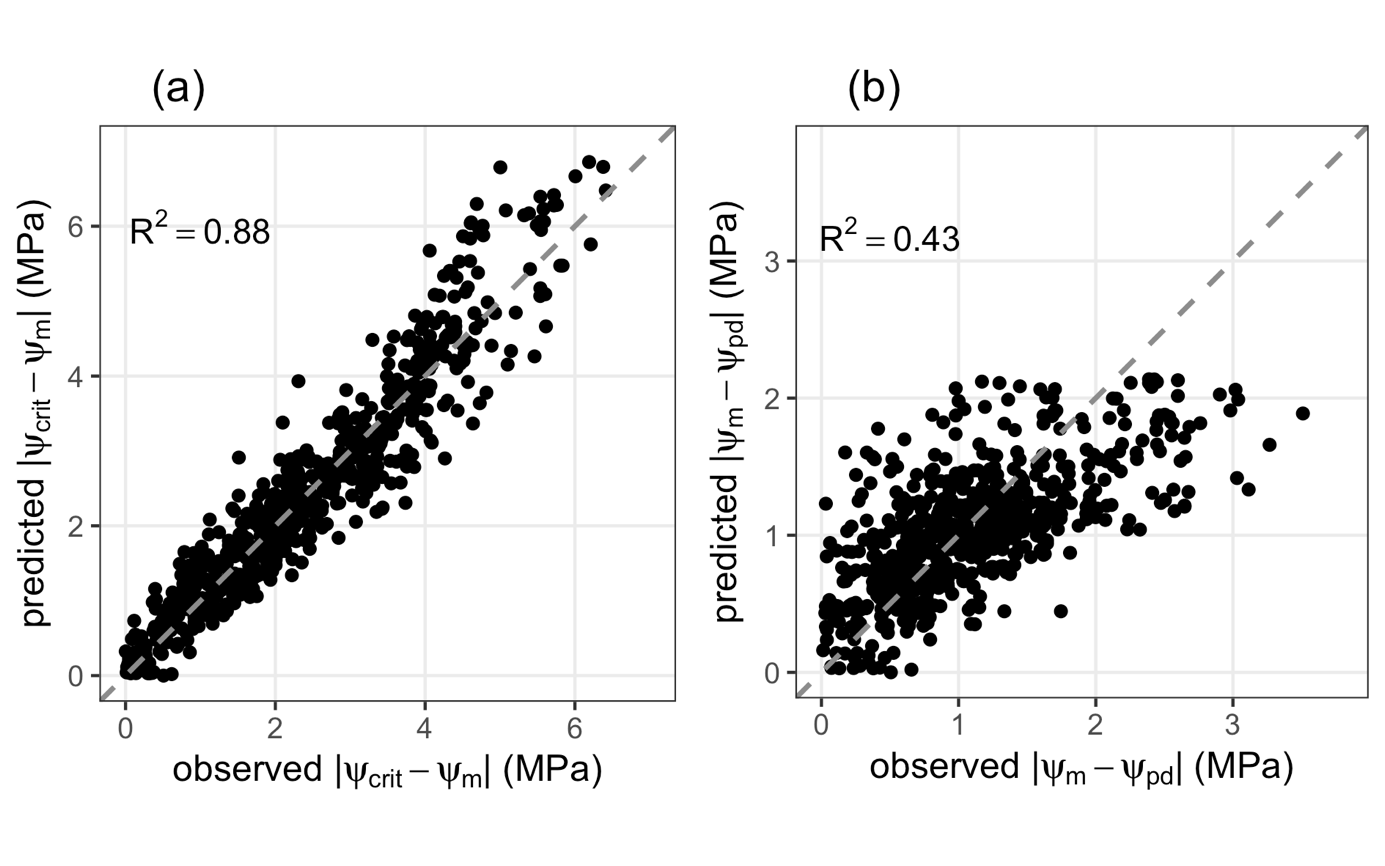


**Supplementary Figure S3** Comparison of specific model predictions, that is, of stress avoidance (a) and water potential gradient maximization (b) with observed data. Data points shown are all data from the dryland water potential database.


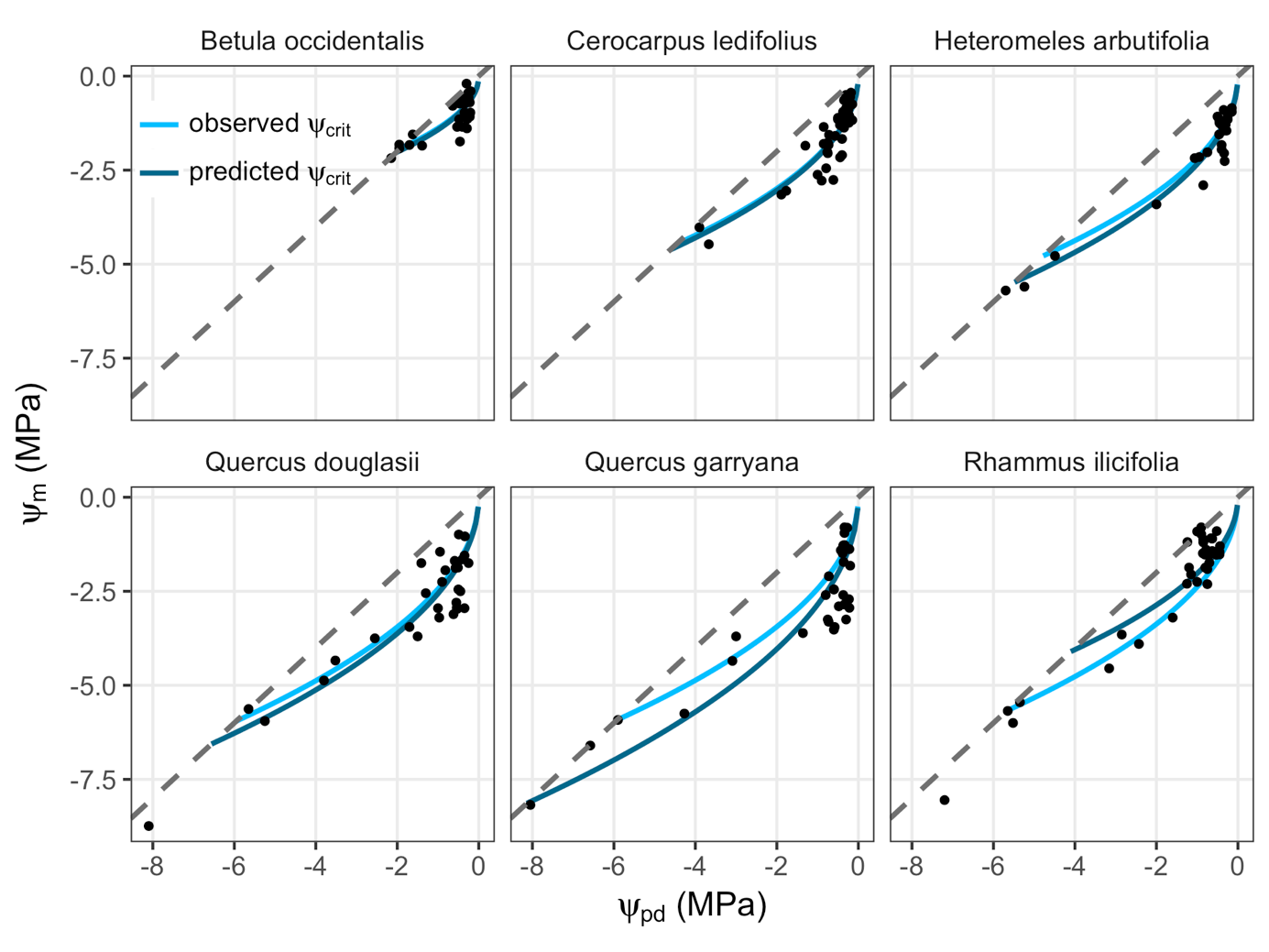


**Supplementary Figure S4** Comparison of predictions of the optimal leaf water status regulation model (solid lines) with measured data^2^ (points) for species not shown in the main article. The model was calculated based on the ψ_crit_ values observed by Meinzer et al.² (light blue lines) and the ψ_crit_ predicted by fitting the model to the data (dark blue lines), grey lines are the one to one line where ψ_pd_ = ψ_m_.


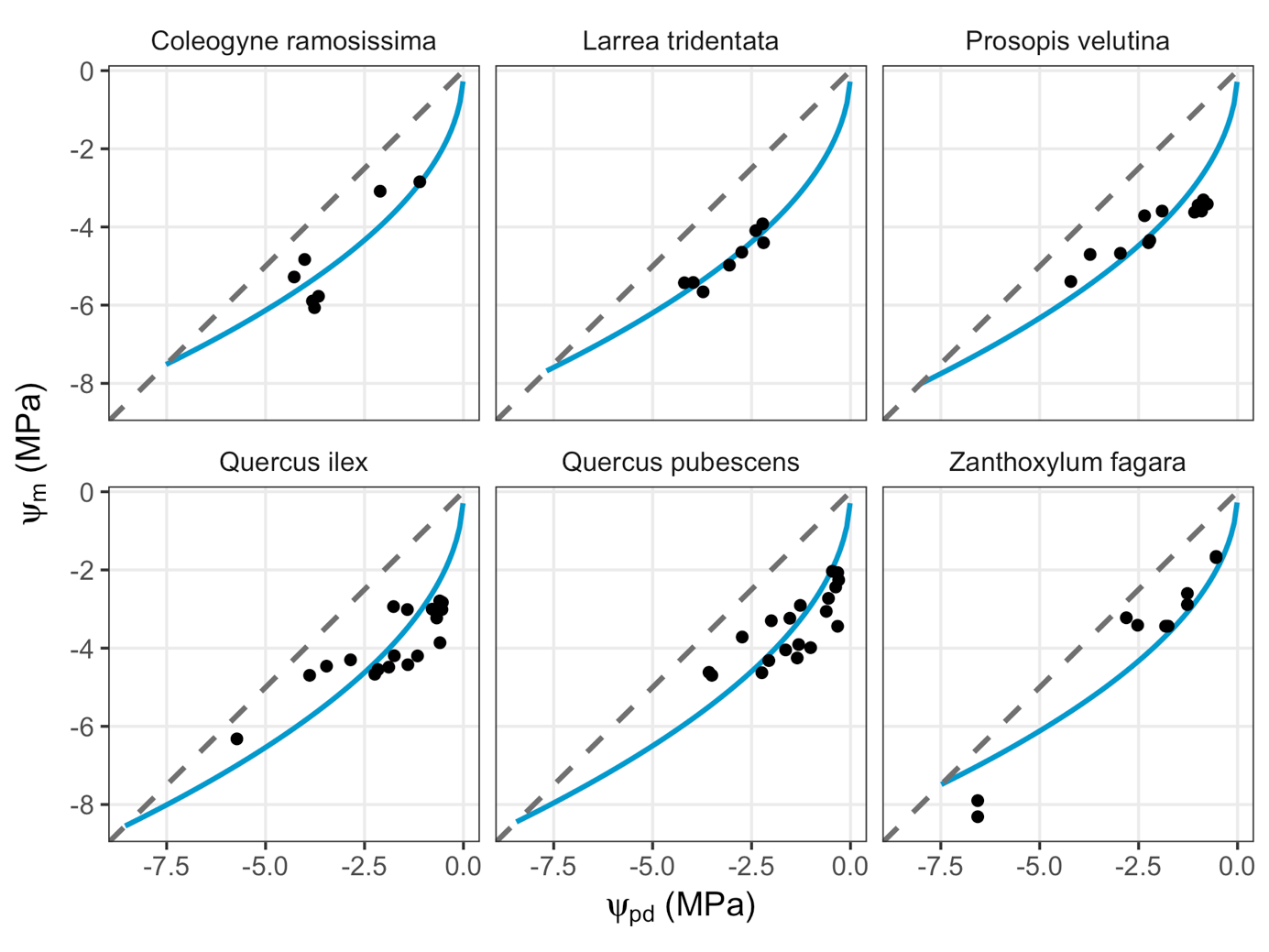


**Supplementary Figure S5** Examples of the optimal leaf water status regulation model (blue solid lines) fitted to datasets in the dryland database for cases where the fit resulted in a ψ_crit_ estimate < -7 MPa not including *Acacia berlandieri*, which is shown in Fig. 2 in the main article. The references can be found in Supplementary Table S3, corresponding IDs are: *Coleogyne ramosissima* 76, *Larrea tridentata* 35, *Prosopis velutina* 73, *Quercus ilex* 52, *Quercus pubescens* 52, *Zanthoxylum fagara* 24.

# **References**

1. Gebrekirstos, A., Teketay, D., Fetene, M. & Mitlöhner, R. Adaptation of five co-occurring tree and shrub species to water stress and its implication in restoration of degraded lands. *Forest Ecol. Manag.* **229,** 259–267 (2006).

2. Meinzer, F. C. *et al.* Mapping ‘hydroscapes’ along the iso- to anisohydric continuum of stomatal regulation of plant water status. *Ecol. Lett.* **19,** 1343–1352 (2016).
